# Transcriptome analysis reveals that long noncoding RNAs contribute to developmental differences between medium-sized ovarian follicles of Meishan and Duroc sows

**DOI:** 10.1038/s41598-021-01817-y

**Published:** 2021-11-18

**Authors:** Mengxun Li, Yi Liu, Su Xie, Lipeng Ma, Zhichao Zhao, Hongbin Gong, Yishan Sun, Tao Huang

**Affiliations:** 1 College of Animal Science and Technology, Shihezi University, Shihezi, 832003 China; 2 Key Laboratory of Bio-Resources and Eco-Environment (Ministry of Education), College of Life Sciences, Sichuan University, Chengdu, 610064 China; 3 Guangxi Yangxiang Animal Husbandry Co. Ltd., Guangxi, Guigang, 537100 China

**Keywords:** Genetics, Molecular biology

## Abstract

Ovulation rate is an extremely important factor affecting litter size in sows. It differs greatly among pig breeds with different genetic backgrounds. Long non-coding RNAs (lncRNAs) can regulate follicle development, granulosa cell growth, and hormone secretion, which in turn can affect sow litter size. In this study, we identified 3554 lncRNAs and 25,491 mRNAs in M2 follicles of Meishan and Duroc sows. The lncRNA sequence and open reading frame lengths were shorter than mRNAs, and lncRNAs had fewer exons, were less abundant, and more conserved than protein-coding RNAs. Furthermore, 201 lncRNAs were differentially expressed (DE) between breeds, and quantitative trait loci analysis of DE lncRNAs were performed. A total of 127 DE lncRNAs were identified in 119 reproduction trait-related loci. In addition, the potential target genes of lncRNAs in cis or trans configurations were predicted. Gene ontology and Kyoto Encyclopedia of Genes and Genomes pathway analysis revealed that some potential target genes were involved in follicular development and hormone secretion-related biological processes or pathways, such as progesterone biosynthetic process, estrogen metabolic process, ovarian steroidogenesis, and PI3K-Akt signaling pathway. Furthermore, we also screened 19 differentially expressed lncRNAs in the PI3K-Akt signaling pathway as candidates. This study provides new insights into the roles of lncRNAs in follicular growth and development in pigs.

## Introduction

Ovulation rate is an important limiting factor of litter size that determines pig reproductive performance, and selection according to the number of ovulations can increase litter size in sows^1^. Differences in ovulation rates may be responsible for differences in litter size between Meishan and Duroc sows. Meishan sows typically ovulate 25–29 eggs per estrus, whereas Duroc sows ovulate an average of only 12.3 eggs^2^. Compared with Duroc sows, Meishan sows have a larger number of medium follicle banks in the follicular phase, and they can maintain a larger number of medium follicle banks in the middle and later stages of the follicular phase. During the follicular phase, the selection period is extended, and more pre-ovulatory follicles are produced that have the potential to discharge greater numbers of mature eggs and give birth to more fetuses^3,4^. Therefore, these two breeds may be considered prolific and ordinary sows, and could be compared to explore variation in follicle development.

Long non-coding RNA (lncRNA) is a type of RNA recently identified in eukaryotes, with length greater than 200 nucleotides but without long reading frames or protein-coding functions^5^. It was initially considered to be the result of noise in the transcription process^6^, but further research has indicated that lncRNAs may be signal molecules, guide molecules, decoy molecules, or scaffold molecules that participate in key regulatory processes in organisms^7^. Furthermore, they have been linked to cancer^8^, immunity^9^, and developmental biology^10,11^. Thus, further studies on lncRNAs could result in significant advancements in our understanding of these fields.

lncRNAs play essential roles in the formation of early germ cells in animals, the implantation and development of early embryos, and the development of reproductive organs. Comparisons of fertilized eggs from humans, mice, cows, and pigs with pre-fertilization gametes and early embryos at different developmental stages through RNA-Seq have detected a large number of lncRNAs related to different embryonic development stages^12–14^. For example, the genes Meg3 and JARID2 interact to recruit PRC2 to inhibit embryonic development-related gene expression in trans^15^. Chen et al. found 24 lncRNAs differentially expressed in the ovaries at different stages of mouse embryonic development and 147 lncRNAs differentially expressed in female and male reproductive organs of the same gestational age^16^. Roeszler found that the lack of lnc-MHM expression in males would hinder the expression of the gonadal testis gene DMRT1, while the lack of expression of lnc-MHM in hens would cause asymmetric ovarian development^17^. However, relatively few studies on lncRNAs in animal ovarian follicle development have been conducted, and there are no relevant studies on pigs.

In this study, we performed high-throughput RNA-seq on RNA samples from medium follicles (M2) of Duroc and Meishan sows. Several lncRNA and mRNA transcripts were detected and differentially expressed lncRNAs and mRNAs between the Meishan and Duroc sows were identified. Gene ontology (GO) and Kyoto Encyclopedia of Genes and Genomes (KEGG) analysis were carried out on the potential target genes (PTGs) of lncRNAs. Some PTGs participate in follicle growth-related biological processes and signaling pathways. Combined with the results of differentially expressed (DE) gene analysis, we found that most of the DE lncRNAs positively regulated the expression of their PTGs. Hence, this study paves the way for further exploring the functions of specific lncRNAs that may be involved in follicular growth and development.

## Results

### Overview of RNA sequencing

We analyzed the RNA data of six libraries of M2 follicles from three Meishan and three Duroc pigs, and obtained 111,275,282 to 142,313,048 raw reads and 101,760,644 to 138,324,398 clean reads per sample, respectively. The clean reads were used after discarding transcripts with adapters, low-quality reads, or other possible contaminants. The Tophat2 software was used to perform reference genomic alignment analysis on clean reads. The mapping ratios of the six sequencing libraries were all greater than 75% (Table 1), indicating that the sequencing accuracy was high and could be used for subsequent analysis.

**Table 1:** The six library samples information.

Sample name	Raw reads	Clean reads	Clean bases	Mapped reads	Mapping ratio	Error rate (%)	Q20 (%)	Q30 (%)	GC content (%)
MFM2DY4_1	111656928	107238580	16.09G	80514662	75.08%	0.02	96.59	91.73	47.14
MFM2DY4_2	121457850	116315790	17.45G	88556504	76.13%	0.02	96.78	92.03	52.18
MFM2DY4_3	111275282	106746570	16.01G	82037293	76.85%	0.02	96.75	91.96	50.09
DFM2DY4_1	118575510	111359682	16.7G	92421276	82.99%	0.02	96.51	91.79	56.43
DFM2DY4_2	107494708	101760644	15.26G	82733818	81.3%	0.02	96.7	92.14	49.84
DFM2DY4_3	142313048	138324398	20.75G	115239405	83.31%	0.02	96.52	91.55	53.96

### Identification and characterisation of lncRNA

**Figure 1:**
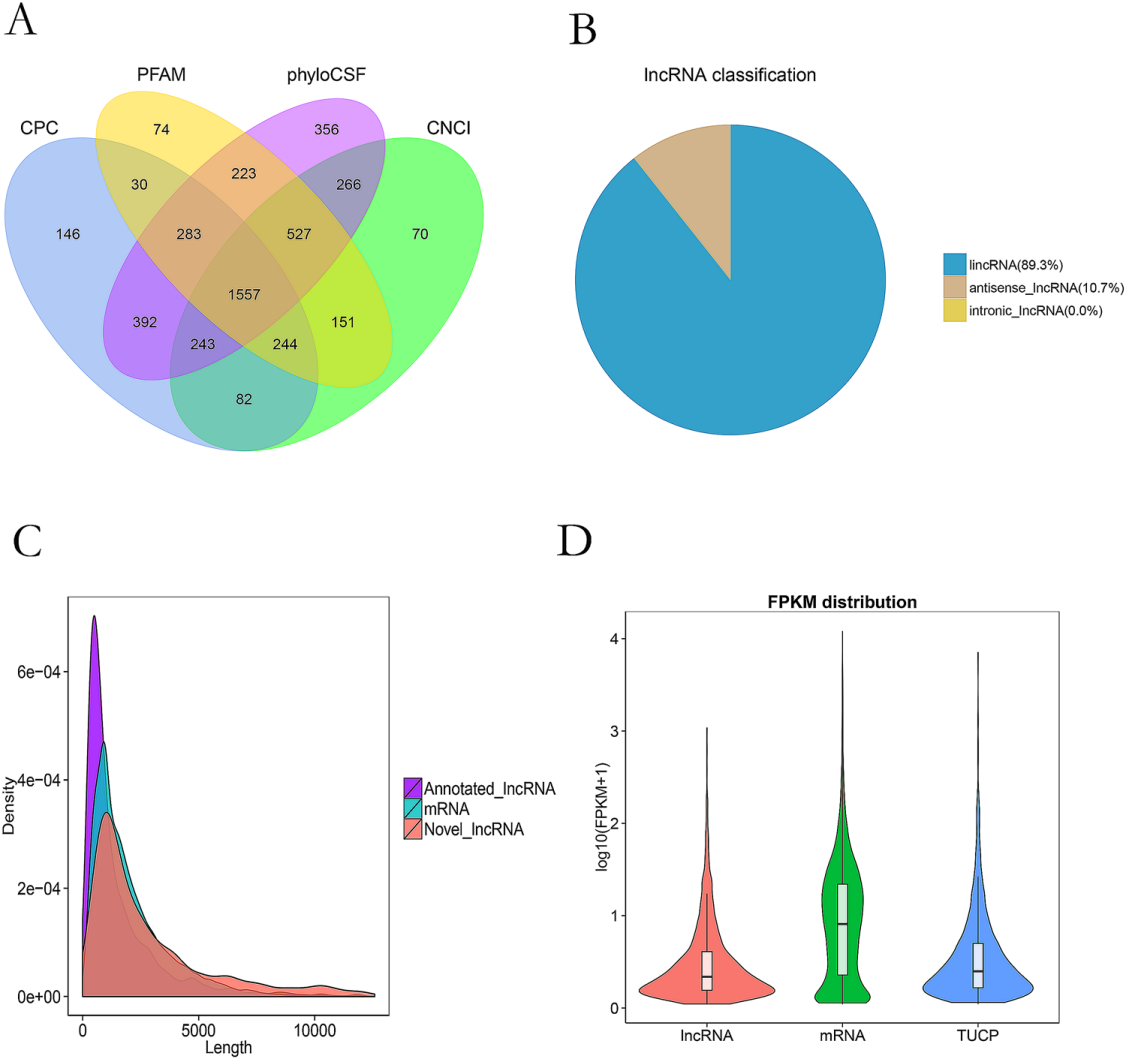
Identification and characterization lncRNAs and mRNAs in M2 follicles. (**A**) Identification of non-coding lncRNAs by using four tools-CPC, PFAM, phyloCSF and CNCI; (**B**) lncRNAs classification; (**C**) The length of lncRNAs and mRNAs; (**D**) Violin plot of expression abundance (showed in log10 (FPKM + 1)) for mRNAs and lncRNAs.

All transcripts were screened in five steps, after which 3554 lncRNAs were identified, including 1997 known lncRNAs and 1557 novel lncRNAs (Fig. 1A, Supplementary Fig. S1). Sense lncRNAs and antisense-lncRNAs accounted for 89.3% and 10.7%, respectively, of the total lncRNAs (Fig. 1B). In addition, we identified 25,491 mRNAs. The lengths of the lncRNA sequences were shorter than those of the mRNAs. The average length of lncRNA transcripts was 1417 bp, while that of the mRNAs was 2423 bp (Fig. 1C). lncRNAs also had fewer exons with exon number ranging from 1 to 10, whereas that of mRNAs ranged from 1 to 30 (Supplementary Fig. S2). Moreover, lncRNAs had shorter ORFs than mRNAs. The ORF lengths of lncRNAs mostly ranged from 1 to 500 bp, whereas those of the mRNAs ranged from 1 to 2000 bp (Supplementary Fig. S3). The expression abundance of lncRNA was lower than that of mRNA (Fig. 1D). The lncRNAs were also less conserved compared to protein-coding genes, as revealed by phastCon analysis (Supplementary Fig. S4). Comparative analysis of the structural features of the selected lncRNAs confirmed the accuracy of selection.

### DE analysis of lncRNAs and mRNAs

**Figure 2:**
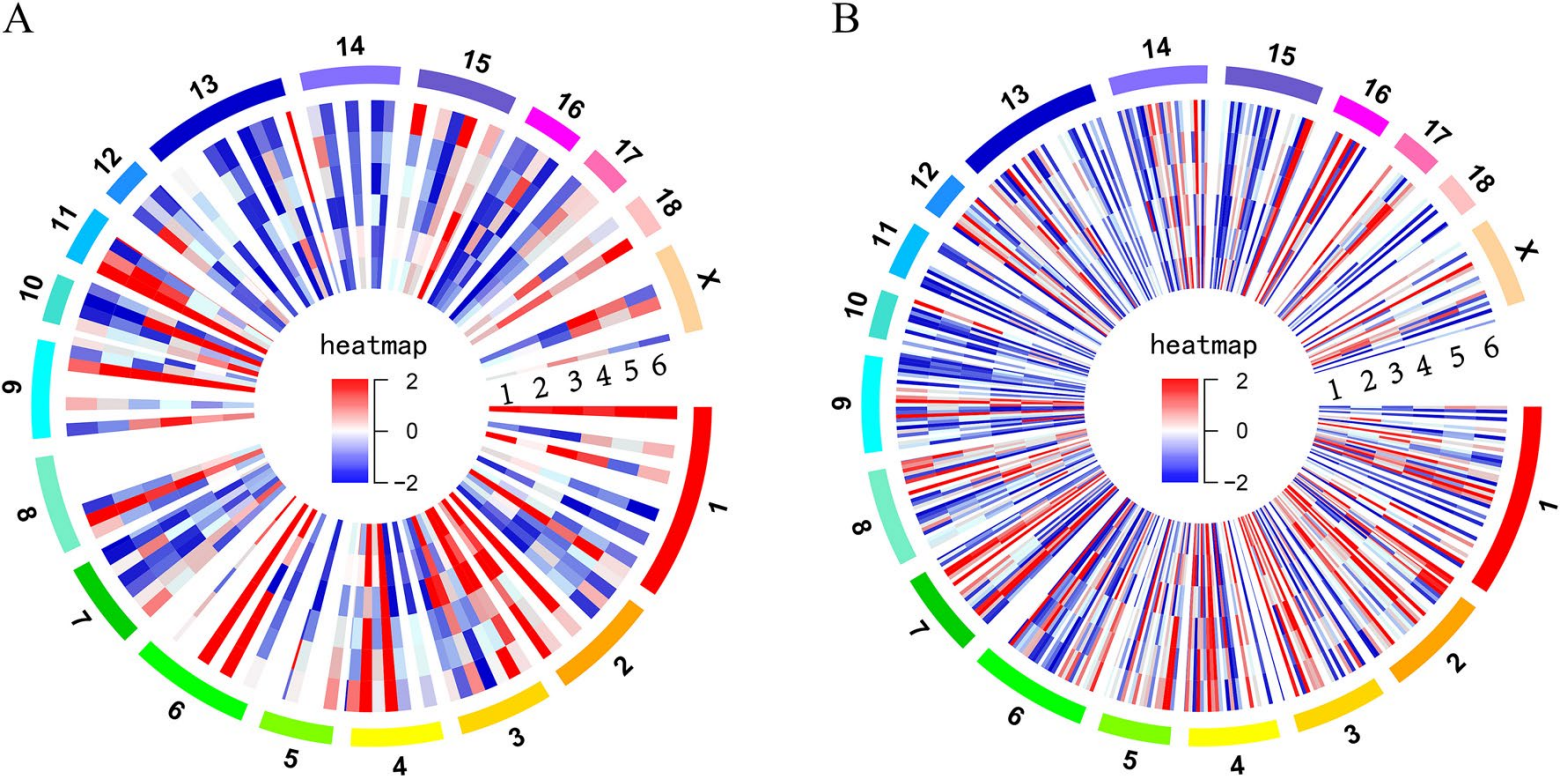
Differential expression (DE) analysis of lncRNA and mRNA in Meishan and Duroc M2 follicle.

**Figure 3:**
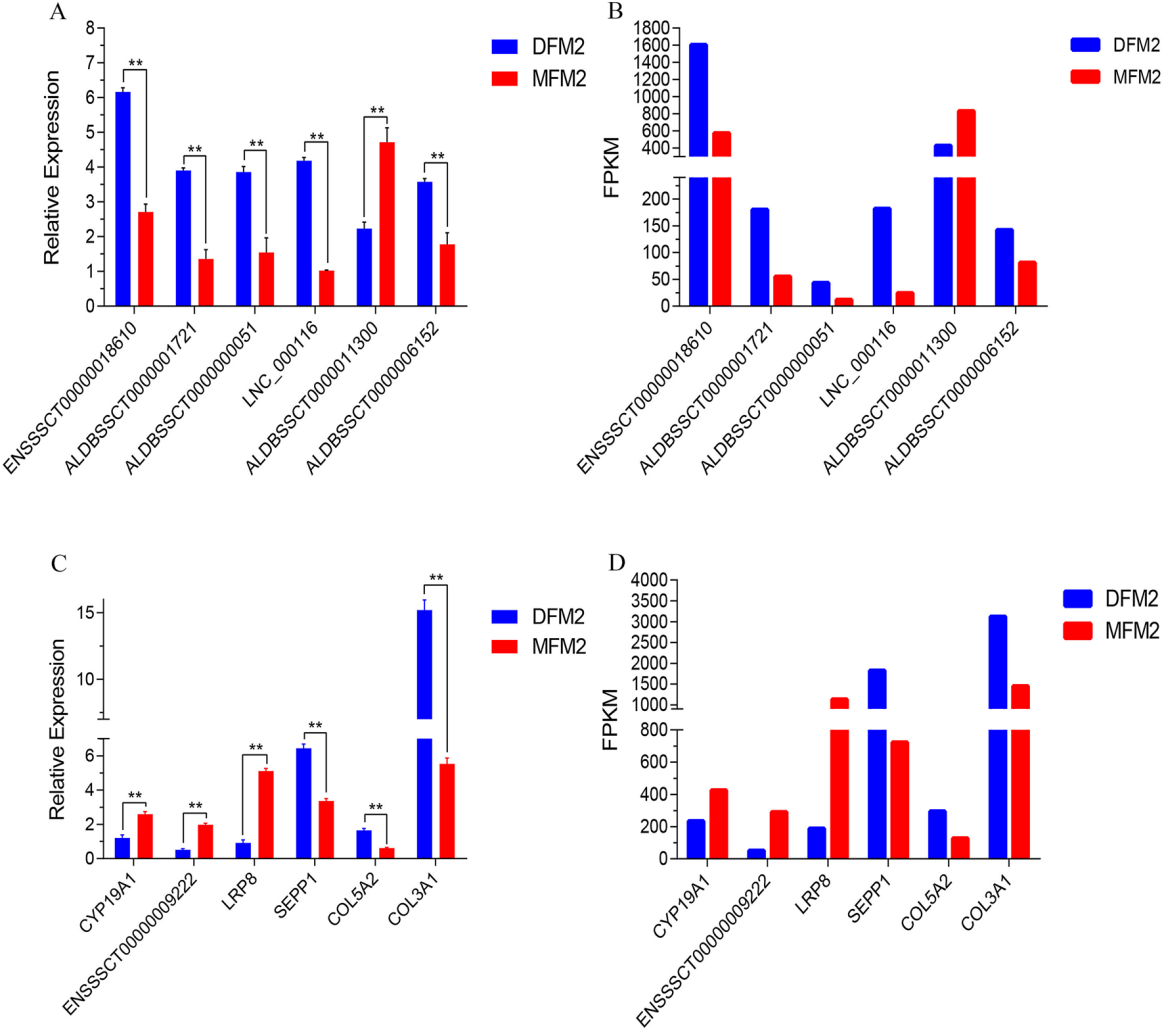
Validation of the expression levels of lncRNAs and mRNAs. The expression of transcripts was normalized by GAPDH. The results were expressed as mean ± SE, *represents P < 0.05, **represents P < 0.01.

We used Cufflinks to normalize transcript expression to FPKM values, and performed differential expression transcript analysis between the Meishan and Duroc medium follicle samples. A total of 201 DE lncRNAs (124 annotated and 77 novol lncRNAs) (p-value adjusted < 0.05, and |log10 FC|> 1) between the two breeds were detected, of which 117 DE lncRNAs were downregulated and 84 were upregulated in Meishan follicles (Fig. 2A). Moreover, 675 DE protein-coding genes were detected, of which 265 were upregulated and 410 were downregulated in Meishan follicles (Fig. 2B). DE lncRNAs and mRNAs were widely distributed on chromosomes, with larger numbers on chromosomes 1, 6, and 13 (Fig. 2A,B). To verify the accuracy of sequencing, six DE-lncRNAs (ENSSSCT00000018610, ALDBSSCT0000001721, ALDBSSCT0000000051, LNC_000116, ALDBSSCT0000011300, and ALDBSSCT0000006152) and six DE-mRNAs (COL3A1, LRP8, ENSSSCT00000009222, SEPP1, COL5A2, and CYP19A1) were selected randomly, and their relative expression levels in the M2 follicles of Duroc and Meishan sows (on day 4) were detected using RT-PCR. The expression analysis of the six lncRNAs and mRNAs is displayed in Fig. 3, which is consistent with the RNA-Seq analysis results for both lncRNA and mRNA (Fig. 3).

### QTL analysis of DE lncRNAs

**Figure 4:**
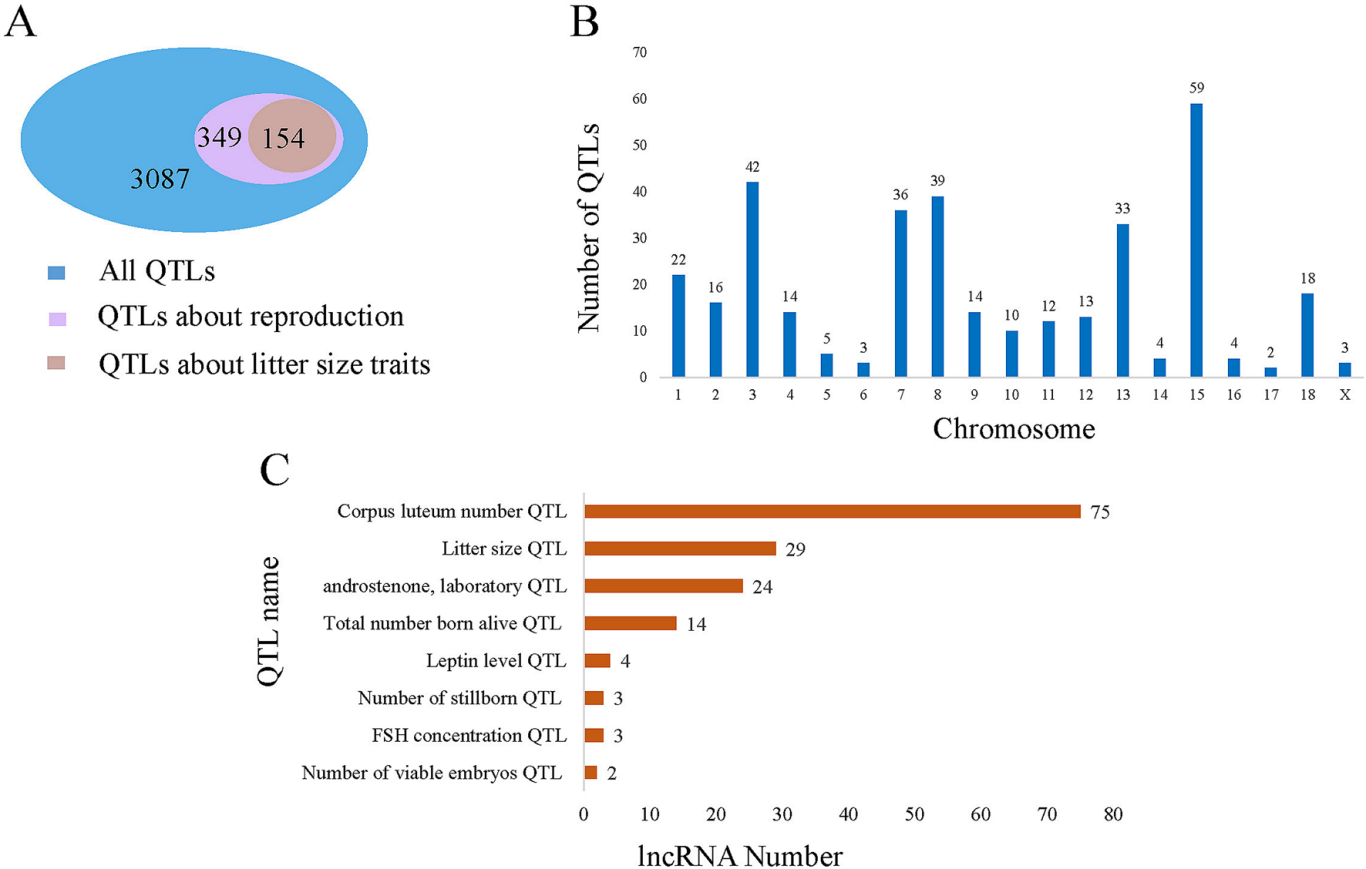
QTLs analysis of DELs. (**A**) The number distribution of QTLs associated with reproduction and all of the QTLs. (**B**) The chromosome distribution of QTLs associated with reproduction. (**C**) The lncRNA number of QTLs associated with reproduction QTLs.

To explore the function of preliminary DE lncRNAs, we mapped the differential expression of lncRNAs transcripts to the QTL database, and performed QTL analysis. The results showed that 1446 QTLs were located in 145 DE lncRNAs, and 127 DE lncRNAs were located in 119 reproduction trait-related loci (Fig. 4A). By studying the distribution of QTLs on the chromosome, 119 QTLs related to reproduction deposition were found to be distributed on chromosomes 1, 2, 3, 4, 7, 8, 13, 15, and 18 (Fig. 4B). These lncRNAs associated with reproduction QTLs could affect corpus luteum number (53 lncRNAs), litter size (18), androstenone (23), total number of live births (4), number of stillborns (3), follicle stimulating hormone concentration (3), and number of viable embryos (2) (Fig. 4C).

### Prediction of lncRNA PTGs

To further explore the regulatory functions of DE lncRNAs, we predicted their PTGs through cis and trans configurations^13,14^. We found that lncRNAs may regulate multiple coding genes. For PTGs regulated by lncRNAs in cis, we searched for protein-coding transcripts located 100 kb upstream or downstream of the DE lncRNAs as its cis-regulatory target genes. We obtained 320 co-localized target genes of 118 DE lncRNAs. In the trans analysis, we used lncRNA-mRNA co-expression analysis and obtained 2,930 PTGs from 127 DE lncRNAs. Here, we only show the PTGs of 24 DE lncRNAs. The number of PTGs for each DE lncRNA was variable. For example, the maximum number of PTGs for an lncRNA was 77 for LNC_000179 and the second highest was 69 for LNC_000715. Some lncRNAs, such as LNC_000718 and LNC_000802, only had two target genes (Table 2).

**Table 2:** Differentially expressed lncRNAs (DELs) and their target genes (PTGs).

Up DELs	Numbers of PTGs			Down DELs	Number of PTGs		
	PTGs	Up regulated	Down regulated		PTGs	Up regulated	Down regulated
ALDBSSCT0000002624	4	4	0	LNC_000179	77	0	77
LNC_000811	15	13	2	LNC_000715	69	68	1
ALDBSSCT0000004325	21	18	3	LNC_001511	29	1	28
LNC_001167	48	48	0	LNC_000846	8	2	6
LNC_000252	36	35	1	LNC_001333	5	1	4
ALDBSSCT0000009309	26	26	0	LNC_000802	2	0	2
LNC_001490	26	10	16	ALDBSSCT0000010765	29	2	27
LNC_000718	2	2	0	ALDBSSCT0000010764	17	0	17
LNC_000460	20	19	1	LNC_000116	49	7	42
LNC_001307	17	10	7	ALDBSSCT0000001721	11	0	11

### Functional enrichment analysis for lncRNAs

**Figure 5:**
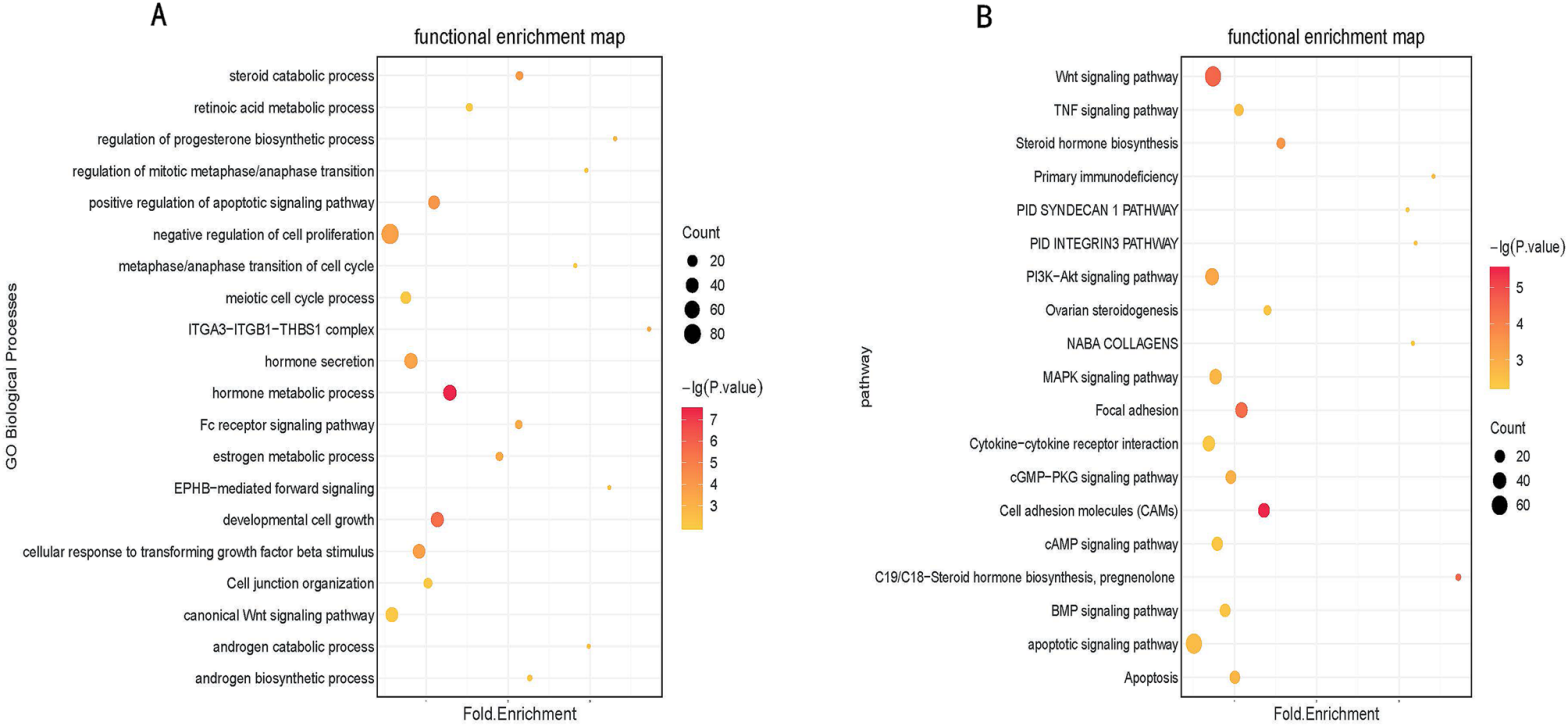
GO and KEGG pathway analysis of the PTGs of differentially expressed lncRNAs (DELs).

To study the regulatory role of DE lncRNAs in Meishan and Duroc M2 follicles, we predicted the function of DE target genes using GO and KEGG to speculate about the functions of lncRNAs. The GO and KEGG analysis results revealed that PTGs participated in 1,063 biological processes and 111 pathways. Many PTGs were involved in biological processes related to follicular development and ovulation, such as the regulation of progesterone biosynthesis, estrogen metabolism, negative regulation of cell proliferation, ITGA3–ITGB1–THBS1 complex formation, cellular response to transforming growth factor beta stimulus, meiotic cell cycle, and steroid catabolic process (p < 0.05) (Fig. 5A). KEGG pathway analysis showed that the PTGs were significantly involved in ovarian steroidogenesis, PI3K-Akt, MAPK, Wnt, BMP, and TNF signaling pathways (p < 0.05) (Fig. 5B).

**Figure 6:**
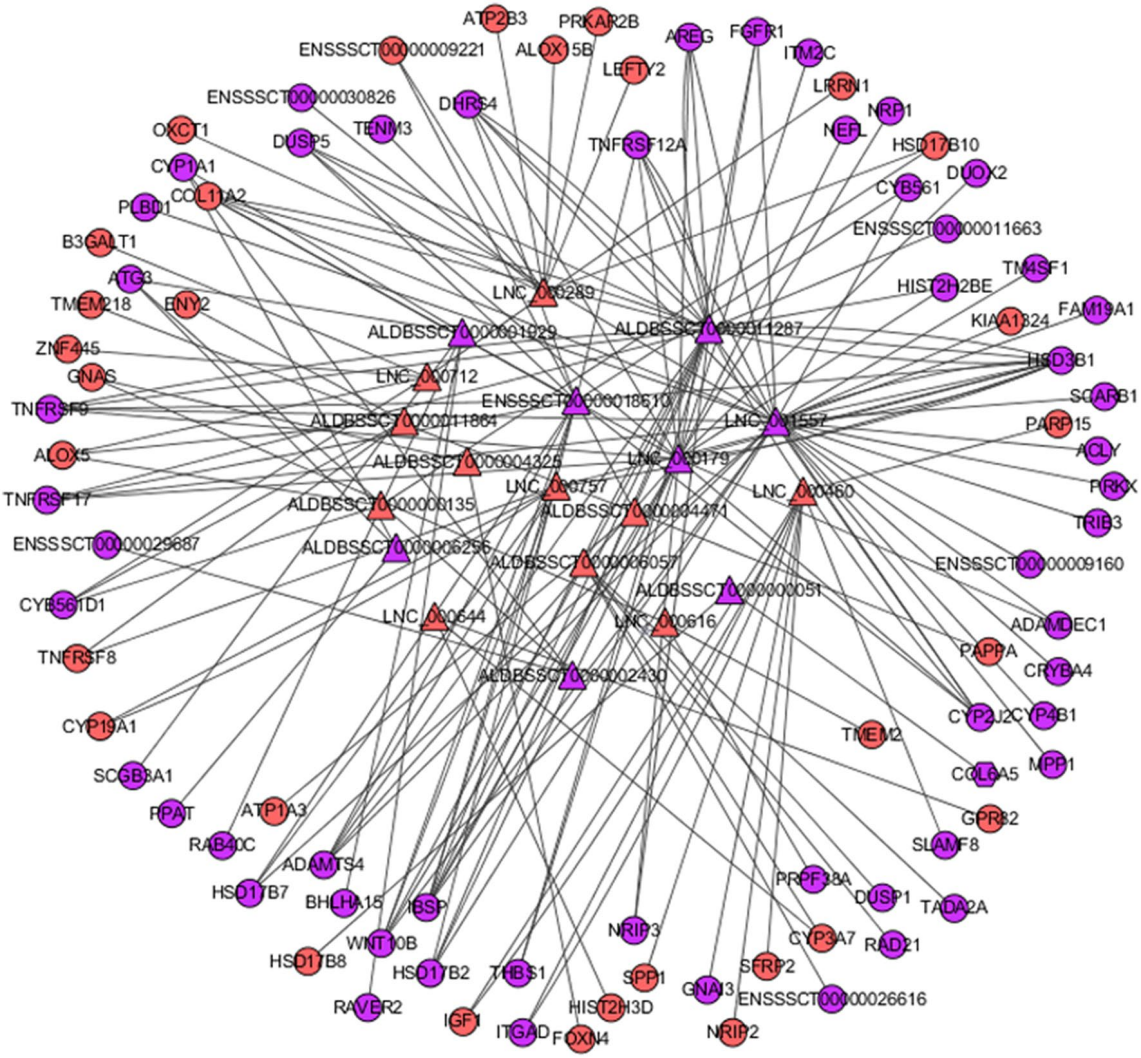
The interaction analysis of PTGs and DELs in estrogen metabolic process and ovarian steroidogenesis signaling pathway. Triangles represent lncRNAs, circles represent mRNA, and red is up-regulated genes, purple is down-regulated genes.

Some PTGs such as CYP1A1, CYP19A1, and HSD3B1 that participate in estrogen metabolic processes and ovarian steroidogenesis signaling pathways are highlighted. For instance, the CYP3A7 gene is involved in the estrogen metabolic process and ovarian steroidogenesis signaling pathway. Similarly, HSD17B8 participates in estrogen metabolism while ALOX5, LHCGR, IGF1, GNAS, CYP2J2, and CYP17A1 participate in the ovarian steroidogenesis signaling pathway. In addition, one protein-coding transcript may be regulated by multiple lncRNAs, such as the DE lncRNAs ALDBSSCT0000001929, ALDBSSCT0000006256, and ALDBSSCT0000002430, which were significantly correlated with their target gene CYP1A1 (p < 0.05). They were all downregulated in Meishan compared to that in Duroc sows. LNC_000644 and ALDBSSCT0000006057 were significantly correlated with CYP3A7 (p < 0.05) and were unregulated in Meishan sows. ALDBSSCT0000000051 was significantly correlated with its target gene, HSD17B8 (p < 0.05). ALDBSSCT0000000051 was downregulated, whereas HSD17B8 was unregulated (Fig. 6). Therefore, we speculated that some DE lncRNAs participate in the development of porcine follicles by positively or negatively regulating their target genes, which are involved in hormone secretion and metabolism.

### Screening of potential reproduction-related lncRNAs in PI3K-AKT signaling pathway in pig ovarian follicle

**Figure 7:**
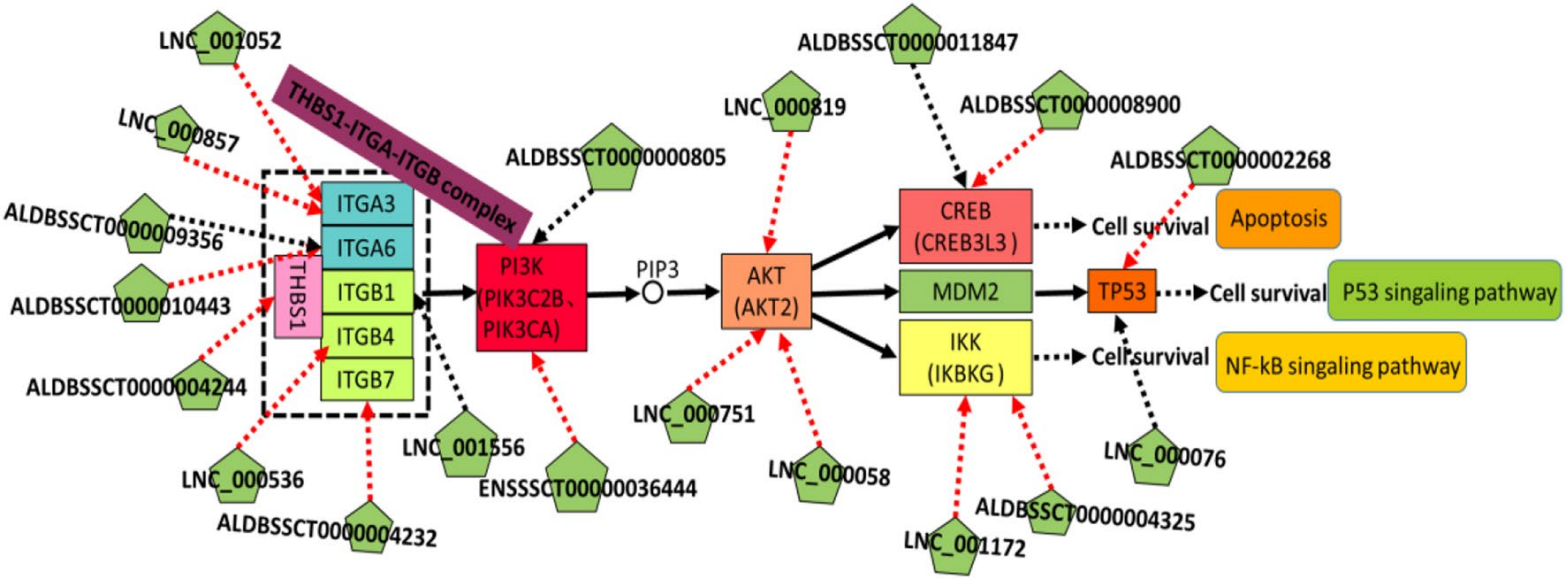
The regulatory network analysis and differentially expressed transcripts involved in PI3K-AKT signaling pathway in our study (RNA-Seq data). The red arrow represents positive correlation, the black arrow represents negative correlation.

The PI3K-AKT signaling pathway is important for porcine follicular development. In order to explore the regulatory roles of the DE lncRNAs involved in ovarian follicle growth and the relationship between these lncRNAs and the PI3K-AKT signaling pathway, we analyzed the expression of lncRNAs and their PTGs in the PI3K-AKT signaling pathway. We found 12 protein-coding genes, namely, THBS1, ITGA3, ITGA6, ITGB1, ITGB4, ITGB7, PIK3C2B, AKT2, CREB3L3, IKBKG, and TP53 in the PI3K-AKT signaling pathway, which were regulated by 19 DE lncRNAs. Some DE lncRNAs and their PTGs showed significant positive correlations (p < 0.05). These included extracellular matrix THBS1 and lncRNA-ALDBSSCT0000004244; ITGA3 and the novel lncRNAs LNC_000857 and LNC_001052; ITGA6 and ALDBSSCT0000010443; ITGB4 and LNC_000536; ITGB7 and ALDBSSCT0000004232; PIK3CA and ENSSSCT00000036444; AKT2 and LNC_000751, LNC_000819 and LNC_000058; CREB3L3 and ALDBSSCT0000008900 and ALDBSSCT000001184; IKBKG and ALDBSSCT0000004325 and LNC_001172; and TP53 and ALDBSSCT0000002268. Some DE lncRNAs and their PTGs were significantly negatively correlated (p < 0.05). These included ITGA6 and ALDBSSCT0000009356; ITGB1 and LNC_001556; PIK3C2B and ALDBSSCT0000000805; CREB3L3 and ALDBSSCT0000011847; and TP53 and LNC_000076 (Fig. 7).

## Discussion

The follicle is a crucial tissue in mammalian reproduction, and its development determines the ovulation rate. Follicle growth and development are complex and precise processes, and lncRNAs play an important role in follicle development. Some studies have elucidated the roles of lncRNAs in animal reproduction through the use of gene chip and sequencing technologies. Macaulay used confocal transmission electron microscopy and RNA-Seq to determine that the cumulus cells around bovine oocytes transported a large number of nutrients and substances, such as mRNAs and lncRNAs, on the oocytes of adult cows^18^. Sun et al. detected 92 DE transcripts between ovulatory follicles of Erhualian and large white pigs using microarrays^19^. Liu identified 2076 lncRNAs and 25,491 mRNAs in ovaries of Duroc sows on days 0, 2, and 4 of follicular growth and development^20^. Meishan and Duroc sows show major differences in ovulation rate and ovarian follicle development and thus are good animal models for uncovering the molecular mechanisms underlying these differences. Therefore, we used RNA-Seq to identify the lncRNAs expressed in the M2 follicles of Meishan and Duroc sows. In our study, the newly identified lncRNAs in pig ovarian follicles have many obvious characteristics; they have shorter and fewer exons, longer exon length, and lower abundance, and they are less conserved than mRNAs. The characteristics of these lncRNAs were consistent with those observed in other studies^21,22^. Thus, the results of this study can be used as a useful resource for conducting further studies on functional lncRNAs and mRNAs in pig ovarian follicles.

We obtained 145 DE lncRNA through QTL analysis, and the results showed that 127 DE lncRNAs were located in QTLs known to determine corpus luteum number, litter size, androstenone, total number born alive, number of stillborns, follicle stimulating hormone concentration, and number of viable embryos. Previous studies have shown that QTLs controlling the ovulation rate are located on pig chromosome 8^23,24^ while QTLs for luteum and ovulation rate are located on chromosome 3^25^. The QTLs of ovulation rate were located on chromosomes 4, 7, 13, and 15 and these QTLs are closely related to pig reproduction^26,27^. In this study, we found that most of the DE lncRNAs, in ovarian follicle lncRNAs had enriched QTLs distributed in chromosomes 3, 7, 8, 13, and 15. These results indicate that DE lncRNAs may regulate porcine reproductive traits and may be related to follicular development in the two pig groups.

lncRNAs have low expression levels, and some lncRNAs have not been clearly identified. Thus, understanding the functions of lncRNAs is difficult. We obtained the target genes of DE lncRNAs, and functional analysis revealed that these target genes participate in multiple biological processes and molecular signaling pathways. CYP19A1 is the target gene of novel lncRNA-LNC_000757, which is expressed in granulosa cells and produces a rate-limiting enzyme for estrogen production. Since the protein from this gene can catalyze the conversion of androgens to estrogens, thereby increasing the expression level of estrogen, knockdown of CYP19A1 can result in the inability to ovulate and lead to loss of corpus luteum in female mice^28^. CYP1A1, an important metabolic enzyme in the estrogen metabolic process and ovarian steroidogenesis signaling pathway, can reduce the level of estrogen and lead to the acceleration of follicular atresia^29,30^. DE lncRNAs ALDBSSCT0000001929, ALDBSSCT0000006256, and ALDBSSCT0000002430 were positively correlated (p < 0.05) with CYP1A1 expression. The expression level of CYP1A1 was lower in Meishan sows, whereas that of CYP19A1 was higher in Meishan than in Duroc sows. These results may indicate that the level of estrogen in the M2 follicles of Meishan sows may be higher than that in Duroc sows. In addition, IGF1 and HSD3B1 can stimulate and promote granulosa cell proliferation and follicular development^31,32^. We also found that HSD17B8, ALOX5, LHCGR, GNAS, CYP2J2, and CYP17A1 participate in the ovarian steroidogenesis signaling pathway. These protein-coding genes may be candidate genes for porcine follicle development and are targeted by one or more lncRNAs (Fig. 6). Therefore, we speculated that lncRNAs may participate in porcine follicular development by regulating their target genes.

Previous studies have shown that the PI3K-AKT signaling pathway plays a major role in ovarian function regulation and follicular development^33–35^. The PI3K-AKT signaling pathway regulates primordial follicle maintenance and activation and promotes apoptosis of ovarian granulosa cells in humans and mice^36,37^. KEGG analysis revealed that some target mRNAs of DE lncRNAs were related to the PI3K-AKT signaling pathway. The members of THBS1-ITGA-ITGB complex members belong to the TGF-β family, the signaling of which regulates the PI3K-Akt signaling pathway. lncRNA-ALDBSSCT0000004 244 targets THBS1, which is an extracellular matrix protein. The expression of THBS1 is driven by estradiol, and its abnormal expression causes apoptosis of granulosa cells and the acceleration of follicular atresia^38^. ITGA3 is the target gene of the novel lncRNAs LNC_000857 and LNC_001052. The expression of ITGA3 in M2 follicles of Meishan sows was higher than that in Duroc sows. However, at present, there are no reports of ITGA3 being related to animal reproduction. The lncRNAs ALDBSSCT0000010443 and ALDBSSCT0000009356 target ITGA6, which may participate in the regulation of cumulus expansion and oocyte maturation^39^. ITGB1, ITGB4, and ITGB7 are the target genes of LNC_001556, LNC_000536, and ALDBSSCT0000004232, respectively. ITGB1 is associated with apoptosis^40^ and is regulated by progesterone and estrogen^41^. The expression of PI3K and AKT is the gold standard for activation of the PI3K-AKT signaling pathway. We found that ENSSSCT00000036444 and ALDBSSCT0000000805 target PIK3CA and PIK3C2B, respectively and LNC_000751, LNC_000819, and LNC_000058 target AKT2. PIK3CA and PIK3C2B are the catalytic subunits of PI3K, and knockout of PIK3CA and PIK3C2B causes early embryo death in mice^42^. PI3K can react with a variety of growth factors, phosphorylating PIP2 to PIP3, activating PDK1, and indirectly or directly activating Akt^43^. AKT2 is a subtype of AKT and a downstream signaling core molecule of the PI3K-AKT classic signaling pathway^44,45^. Activated AKT2 can phosphorylate its downstream signaling molecules and produce cAMP, which can activate CREB, TP53, and IKK protein for nuclear gene transcription and expression, thereby regulating cell proliferation^46,47^. ALDBSSCT0000011847 and ALDBSSCT0000008900 target CREB3L3. ALDBSSCT0000004325 and LNC_001172 target the IKBKG, TP53 and ALDBSSCT0000002268 and were both significantly positively correlated in pairs (p < 0.05), LNC_000076 and ALDBSSCT0000002268 target TP53. CREB3L3 is a transcription factor of CREB, and its function is similar to that of CREB. Its abnormal expression can attenuate the upregulation of Egr1 by GnRH receptor activation, which in turn affects the expression level of LH-β and the growth of granular cells^48^. IKK can induce phosphorylation of IkB (inhibitory protein of NF-κB), dissociation of the NF-kB/IkB dimer, activation of NF-Kb, inhibition of the apoptosis pathway, and maintenance of porcine follicular development^49^. TP53 is a critical factor for cell survival, and suppression of p53 in oocytes can promote follicular growth and development^50^. In this study, we screened 19 DE lncRNA candidates and their 12 target genes in the PI3K-AKT signaling pathway. We suggest that lncRNAs may participate in follicular growth and development by regulating their target genes, but the specific functions require further research.

## Methods

### Animals and sample collection

All experiment related to animal work in our study were carried out according with the guidelines of the Institutional Animal Care and Use Committee (IACUC) at the Medical Ethics Committee, First Affiliated Hospital, Medical College, Shihezi University(Approval Number: 2014-073-01, March 5, 2014). Three multiparous Meishan and three multiparous Duroc cyclic sows were raised at the Animal Experiment Station of Shihezi University. The sows were observed every day to determine their natural estrus cycle (the first day of estrus was considered day 0). Sows were injected with PGF2α according to the weight of the pigs measured by an analog weighing scale (cloprostenol, Ningbo Second Hormone Factory) on the 14th day of the estrus cycle to induce luteal regression and to synchronize the follicular growth phase. Four days later, the sows were subjected to electric stunning and quick bleeding, and then, the ovaries and medium follicles (M2 follicle diameter: 5.0–6.9 mm) were harvested, which was recorded as MFM2DY4_1, MFM2DY4_2, MFM2DY4_3 and DFM2DY4_1, DFM2DY4_2, DFM2DY4_3, and take three M2 follicles from the ovaries of each sow. Each M2 follicles samples were frozen in liquid nitrogen, and stored at – 80 °C in a refrigerator until RNA isolation.

### RNA-Seq preparation and sequencing analysis

Total RNA from all follicle samples was extracted using the TRIzol reagent (Invitrogen). RNA degradation and contamination were monitored using 1% agarose gels. The total RNA concentration, integrity, and purity were detected using a qubit RNA assay kit in Qubit 2.0 Fluorometer, RNA Nano 6000 assay kit in Bioanalyzer 2100 (Agilent Technologies), and a NanoPhotometer spectrophotometer (IMPLEN). The ribosomal RNA of all samples was removed using a Ribo-zero rRNA Removal Kit (Epicenter). Six strand-specific RNA-Seq libraries were constructed from the M2 follicles of the six sows according to the manufacturer’s instructions. The sample library fragments were purified using the AMPure XP system (Beckman Coulter, USA) to extract the preferred cDNA fragments of 150–200 bp. The blunt-end cDNA fragments were augmented with an A base and ligated to the sequencing adapter. The final products were purified (AMPure XP System), and the quality of the library was assessed using the Agilent Bioanalyzer 2100 system. The samples were then analyzed using one lane of 100–200 nt paired-end HiSeq 4000 platform. Quality control (QC) of RNA-Seq reads was performed using Fast QC.

### Transcriptome assembly

We filtered reads with adapters and low-quality poly-*N* reads from raw data through in-house Perl scripts to obtain clean reads. These high-quality clean reads were used for all subsequent analyses. The pig reference genome annotation index was built using Bowtie v2.0.6^51^. Appropriate parameters were set using Tophat2 v. 2.0.9^52^. Scripture and Cufflinks^53^ were used to assemble and splice aligned sequences, which can be as small as possible. The transcript set, Cufflinks, has specific parameters for the chain-specific library, and the directional information of the transcript chain was accurately provided.

### lncRNA identification

The following steps were used to identify lncRNAs from the non-redundant transcriptome. (1) Transcripts with a single exon or two exons were filtered out. (2) Transcripts with length < 200 bp were removed. (3) Any transcript with fragment per kilobase of transcript per million mapped reads (FPKM) score lower than 0.5 in every sample was discarded. (4) The remaining transcripts were blasted in the known pig annotation lncRNA database (ALDB)^54^ using Cuffcompare. Only the transcripts of lncRNAs with splice sites congruent between our results and ALDB were taken as known lncRNAs. (5) Transcripts of any known protein-coding sequences were discarded, and transcripts that belonged to pre-miRNA, snRNA, rRNA, snoRNA, and pseudogenes were removed. (6) The Coding-Non-Coding Index (CNCI), Coding Potential Calculator (CPC), and phyloCSF tools were used to calculate transcripts with coding potential. Transcripts with CNCI score < 0^55^, CPC score < 0^56^, Pfam-scan E-value < 0.001^57^, and phyloCSF Max-score ≤ 100^58^, as well as the intersection of these tools were defined as novel lncRNA transcripts.

### Differentially expressed lncRNAs and mRNA analysis

We used the Cuffdiff utility provided by the Cufflflinks package to conduct differential expression tests between two breeds. The Cufflinks package was used to conduct DE analysis between six follicle libraries of the Meishan and Duroc sows. The fold change (FC) values were calculated as log10 (FPKM_MFM2/FPKM_DFM2) (FPKM_MFM2: FPKM of Meishan M2 follicle; FPKM_DFM2: FPKM of group Duroc M2 follicle). Transcripts with adjusted p-values < 0.05 were identified as differentially expressed.

### RT-PCR verification

Total RNA was extracted using TRIzol (Invitrogen, CA, USA) and cDNA was synthesized using an RT-PCR kit (TaKaRa, Japan), and RT-PCR was performed using SYBR Green I (TaKaRa Biotech, Dalian, China) according to the manufacturer’s protocol. The reaction was conducted by combining 12.5 µL of 2 × Real Master Mix (TaKaRa Biotechnology), 2 μL of cDNA, 1 μL each of the upstream and downstream primers, and 8.5 μL of RNase-free ultra-pure water. Specific primers were designed using the Primer Premier 5.0 (Table 3) and confirmed with BLAST. The expression levels of genes were normalized to linear GAPDH levels using the 2(− ΔΔCt) method^59^, and statistical differences were analyzed using SPSS ver. 17.0. The correlation between the results of RNA-Seq and RT-PCR was calculated using a correlation test.

**Table 3:** The specific primers for RT-PCR.

Transcript type	Transcript name	Forward primer	Reverse primer	Product size (bp)
LncRNA	ENSSSCT00000018610	TGGTCTGCTCTAACCTGGACT	CTTCAGACAGGCTCAAGGGG	297
	ALDBSSCT0000001721	ACTCTTCAGTGGAGCTGACAA	TGGTCAAATTTTCCCTGGGATTG	81
	ALDBSSCT0000000051	AAGCAGAGGACGAAAAAG	CTACGCCACTCCAGAAAG	128
	LNC_000116	GCCTCTCTTGGTGCTTGGTT	TCGGTGGCTTCGGAGTTATT	134
	ALDBSSCT0000011300	CAAGGGGGTCAATTTTGCC	CACGCCTTGTGAATCGGTTT	122
	ALDBSSCT0000006152	AGTTCTCCAATGTCCCGTGTC	AGAAGACGCAGCCATCGGA	241
mRNA	COL3A1	ATCGCTGGTGTTGGAGGT	GAAGTCATAATCTTGTCGGTGT	100
	LRP8	CCAATCGCATCTACTGGTGTGAC	GGAGAGTGCAGCTGCTCATCAAT	115
	ENSSSCT00000009222	ATGCCTTCAATGGGACAACG	CAGTGGCTGGGGTAAGTCAA	262
	SEPP1	CCTTCATTGACCTCCACTAC	GTTGTCATACTTCTCATGGTTC	320
	COL5A2	GGGACATTTGGAAACCTGCC	GGGGAGTTATGGGGTCAGCA	114
	CYP19A1	CCAGCATTACCAGAAGCC	TGTGCCTCCATTACCGAG	92
	GAPDH	TTCCAGTATGATTCCACCCACG	TCGGCAGAAGGGGCAGAGAT	242

### QTL analysis of DE lncRNAs

To predict the functions of DE lncRNAs, QTL analysis was performed. Firstly, the location information of DE lncRNAs was obtained from the transcriptome file. Secondly, we download the pig quantitative trait locus (QTL) database (Pig QTLdb: https://www.animalgenome.org/cgi bin/QTLdb/SS/download/ bedSS 11.1). Next, BEDTools and the “intersectBed” command were used for QTL analysis^60^, and obtain all differential expressions from the only transcriptome file Location information of lincRNA. Finally, we use the BEDTools2.17.0 tool to perform DEL mapping and find the corresponding QTL.

### Prediction of PTGs of lncRNAs

Two methods were used to predicte the PTGs of lncRNAs. We identified the PTGs regulated by lncRNAs in cis, which were defined as protein-coding genes located at 100 Kb upstream and downstream of the lncRNA, by BEDTools 2.17.0^61^. The trans regulation of a lncRNA and its PTG was identified by the expression level correlation analysis of each pair of lncRNA and PTG. According to the Pearson’s correlation coefficients (|r|> 0.95), the PTGs were selected to construct a lncRNA–mRNA co-expression network.

### GO and KEGG pathway enrichment analysis

The prediction of the lncRNA and PTGs’ function such as gene enrichment and pathway analysis were performed using gene ontology (GO) (http://www.geneontology.org/)^62^ and Kyoto Encyclopedia of Genes and Genomes (KEGG) (http://www.genome.jp/kegg)^63^. To further explore the main biological functions of differentially expressed genes, KOBAS software was used to detect the statistical enrichment of lncRNA PTGs or DEGs in KEGG pathways. The enrichment findings with a q-value of less than or equal to 0.05 was considered statistically significant.

### Ethics approval and consent to participate

All slaughtering, sample collection and experimental related to animal work in our study were carried out according with the guidelines of the Institutional Animal Care and Use Committee (IACUC) at the Medical Ethics Committee, First Affiliated Hospital, Medical College, Shihezi University (Approval Number: 2014-073-01, March 5, 2014). The experimental protocol in our study was approved by the Institutional Animal Care and Use Committee (IACUC) at the Institute of the Medical Ethics Committee, First Affiliated Hospital, Medical College, Shihezi University. The study was carried out in compliance with the ARRIVE guidelines.

## Supplementary Information

Supplementary Figure S1.

Supplementary Figure S2.

Supplementary Figure S3.

Supplementary Figure S4.

## Data Availability

The data and material required to reproduce these findings can be shared at this time as the data also forms part of an ongoing study. The original data has been uploaded to the NCBI database, number: SRR16047914.

## Abbreviations

lncRNA: Long non-coding RNAs
DE: Differentially expressed
GO: Gene ontology
KEGG: Kyoto encyclopedia of genes and genomes
PTG: Potential target gene
CNCI: Coding-non-coding index
CPC: Coding potential calculator
QTL: Quantitative trait locus
FPKM: Fragments per kilobase per million
QC: Quality control
FC: Fold change
RT-PCR: Real-time PCR
PI3K-Akt: The phosphatidylinositol 3′-kinase-Akt
MAPK: Mitogen-activated protein kinase
*HSD3B1*: 3-Beta-hydroxysteroid dehydrogenase type 1
*ALOX5*: Arachidonate-5-lipoxygenase
*THBS1*: Thrombospondin 1
ITGB1: Integrin subunit beta 1
TGF-β: Transforming growth factor-β
