# Supplementary figures and images for: Transcriptome analysis reveals that long noncoding RNAs contribute to developmental differences between medium-sized ovarian follicles of Meishan and Duroc sows
Source: Sci Rep. 2021 Nov 18;11:22510. doi: 10.1038/s41598-021-01817-y (PMC8602415; doi:10.1038/s41598-021-01817-y)

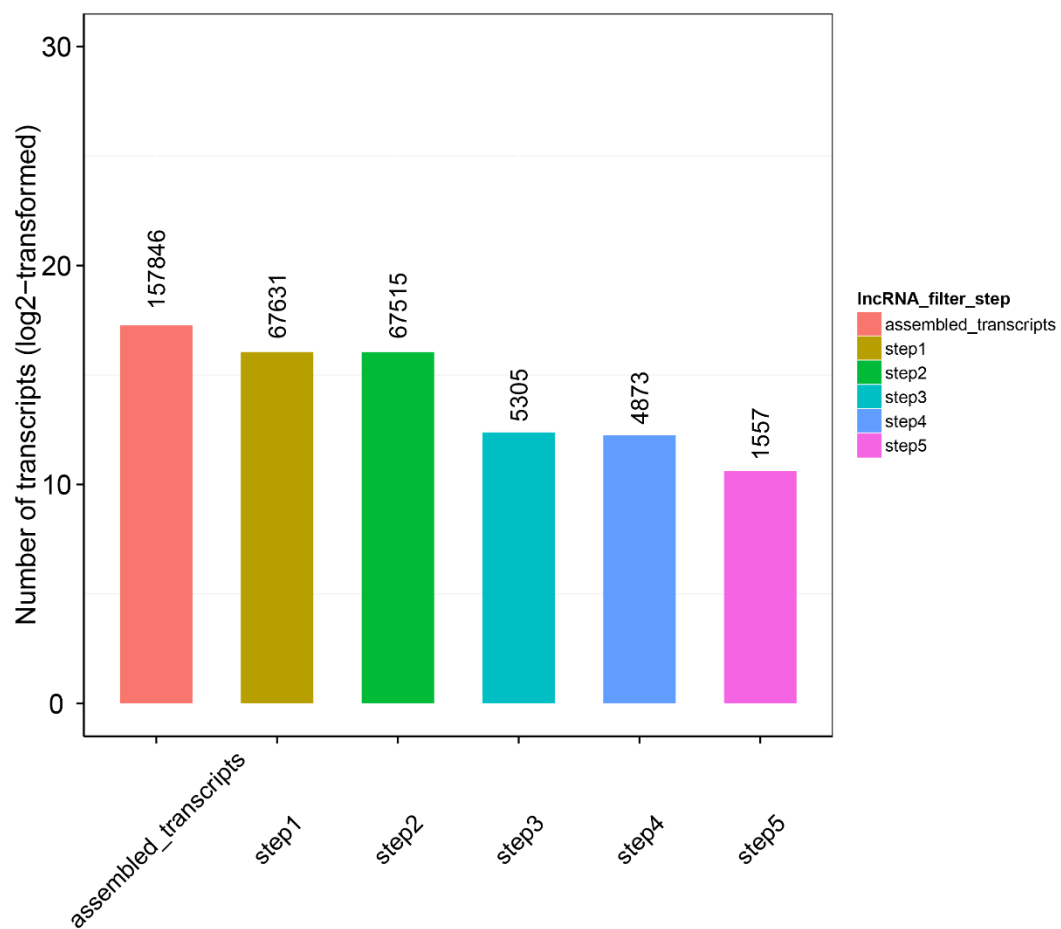

Fig. S1. lncRNA\_filter\_stat, 157, 846 transcripts were assembled

Supplement: Supplementary file 1 — Supplementary Figure S1. [file 41598_2021_1817_MOESM1_ESM.pdf]

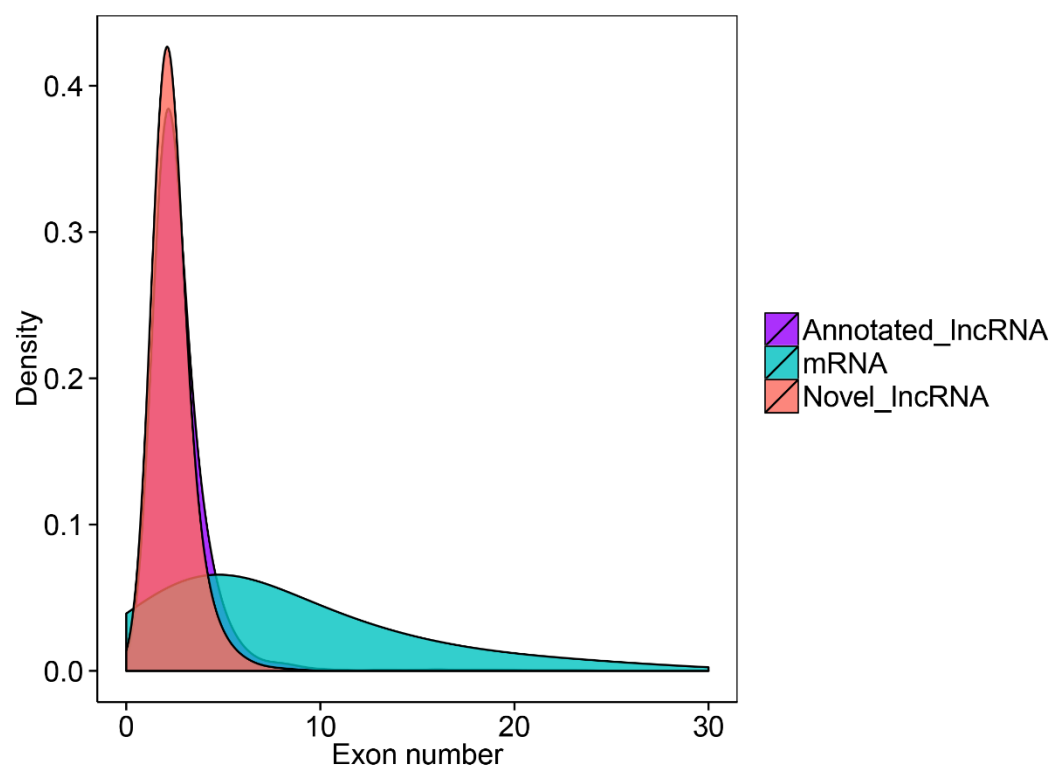

Fig. S2. The lncRNAs and mRNAs exon number

Supplement: Supplementary file 2 — Supplementary Figure S2. [file 41598_2021_1817_MOESM2_ESM.pdf]

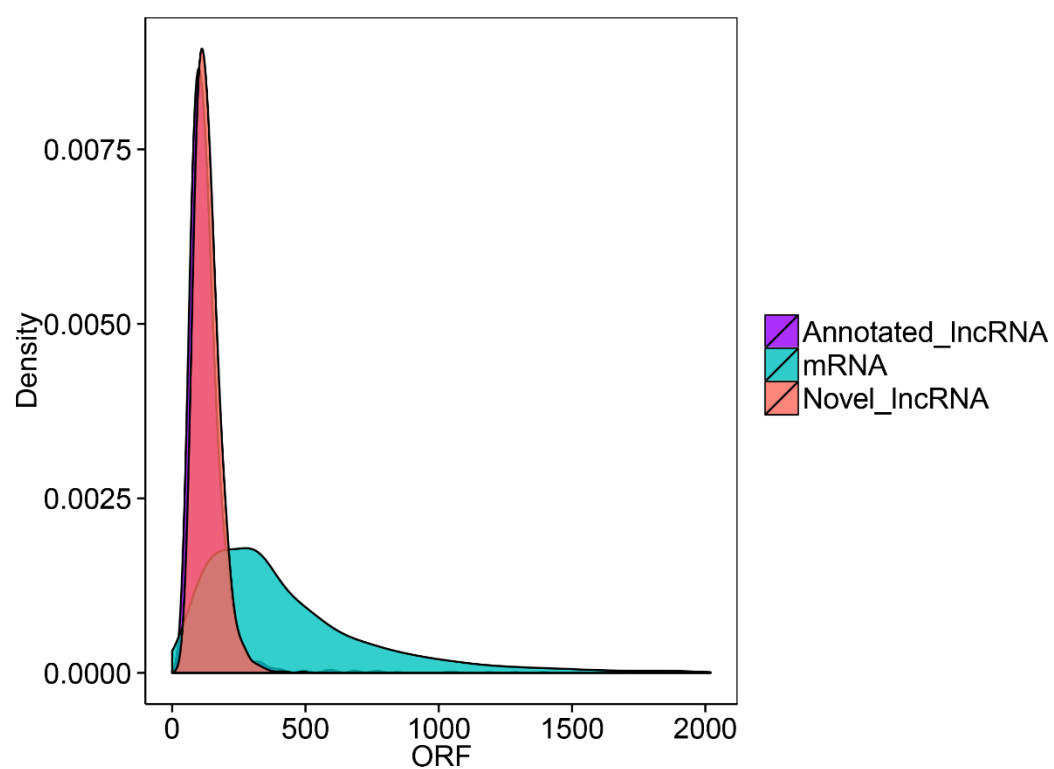

Fig. S3. Open reading frame (ORF) length distribution of mRNAs and lncRNAs

Supplement: Supplementary file 3 — Supplementary Figure S3. [file 41598_2021_1817_MOESM3_ESM.pdf]

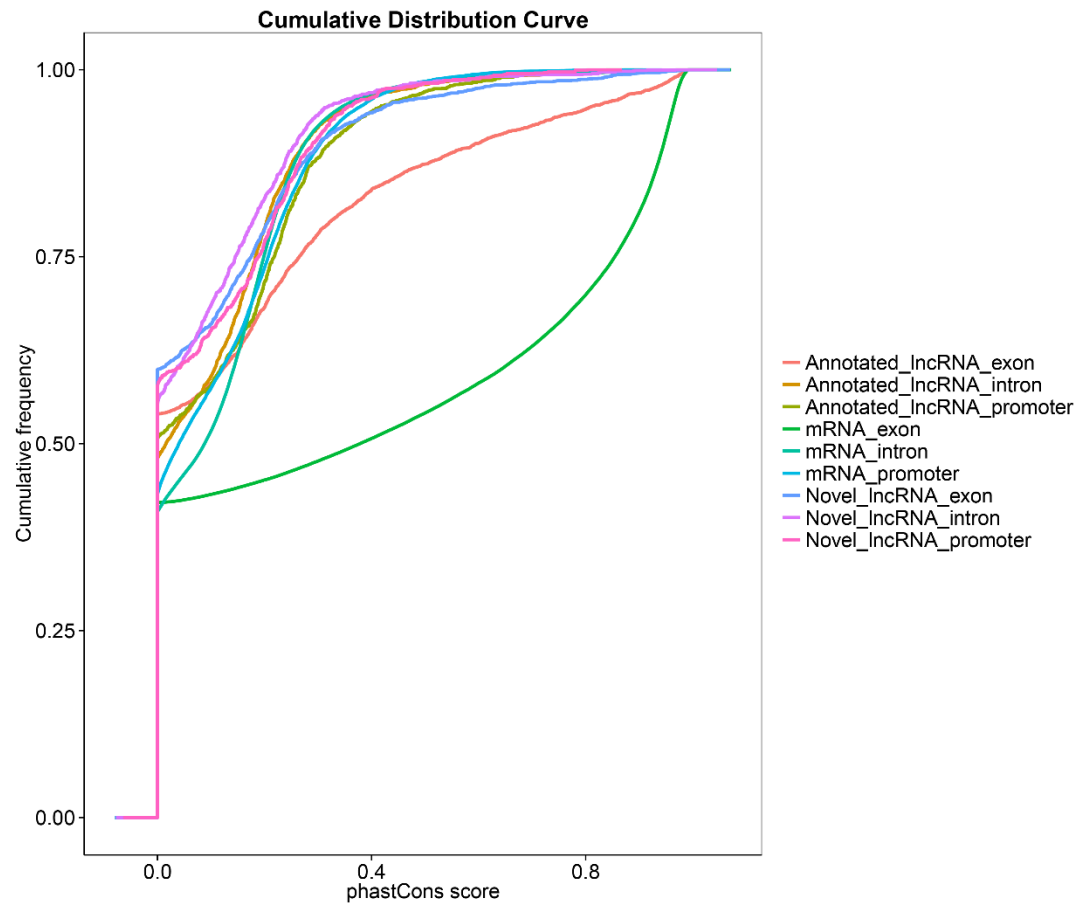

Fig. S4. Conservation compare with mRNAs and lncRNAs by using phasConsoftware

Supplement: Supplementary file 4 — Supplementary Figure S4. [file 41598_2021_1817_MOESM4_ESM.pdf]
